# Genome Copy Number Quantification Revealed That the Ethanologenic Alpha-Proteobacterium *Zymomonas mobilis* Is Polyploid

**DOI:** 10.3389/fmicb.2021.705895

**Published:** 2021-08-02

**Authors:** Katsuya Fuchino, Daniel Wasser, Jörg Soppa

**Affiliations:** ^1^Institute for Molecular Biosciences, Goethe-University, Frankfurt, Germany; ^2^Department of Biotechnology and Food Science, NTNU Norwegian University of Science and Technology, Trondheim, Norway

**Keywords:** genome copy numbers, polyploidy, *Zymomonas mobilis*, *alpha-proteobacteria*, chromosome organization, qPCR, cell volume

## Abstract

The alpha-proteobacterium *Zymomonas mobilis* is a promising biofuel producer, based on its native metabolism that efficiently converts sugars to ethanol. Therefore, it has a high potential for industrial-scale biofuel production. Two previous studies suggested that *Z. mobilis* strain Zm4 might not be monoploid. However, a systematic analysis of the genome copy number is still missing, in spite of the high potential importance of *Z. mobilis*. To get a deep insight into the ploidy level of *Z. mobilis* and its regulation, the genome copy numbers of three strains were quantified. The analyses revealed that, during anaerobic growth, the lab strain Zm6, the Zm6 type strain obtained from DSMZ (German Collection of Microorganisms), and the lab strain Zm4, have copy numbers of 18.9, 22.3 and 16.2, respectively, of an origin-adjacent region. The copy numbers of a terminus-adjacent region were somewhat lower with 9.3, 15.8, and 12.9, respectively. The values were similar throughout the growth curves, and they were only slightly downregulated in late stationary phase. During aerobic growth, the copy numbers of the lab strain Zm6 were much higher with around 40 origin-adjacent copies and 17 terminus-adjacent copies. However, the cells were larger during aerobic growth, and the copy numbers per μm^3^ cell volume were rather similar. Taken together, this first systematic analysis revealed that *Z. mobilis* is polyploid under regular laboratory growth conditions. The copy number is constant during growth, in contrast to many other polyploid bacteria. This knowledge should be considered in further engineering of the strain for industrial applications.

## Introduction

For decades it was thought that bacteria and archaea are typically monoploid, with very few exceptions like the very radiation-resistant species *Deinococcus radiodurans*. However, during recent years it has become clear that many bacterial species are oligoploid (up to 10 genome copies), polyploid (up to 100 genome copies), or even hyperpolyploid. Oligo-/polyploid species have been identified in various phylogenetic groups, e.g., in proteobacteria (Pecoraro et al., 2011), cyanobacteria (Griese et al., 2011), gram-positive bacteria (Böttinger et al., 2018), methanogenic archaea (Hildenbrand et al., 2011), and halophilic archaea (Breuert et al., 2006). In addition, all giant bacteria appear to be polyploid (Angert, 2012). Various phylogenetic advantages of polyploidy for prokaryotes have been described, and it is thought that during evolution various species in different phylogenetic groups developed oligo-/polyploidy at different times for different reasons (Soppa, 2014; Ludt and Soppa, 2019). Phylogenetic groups and subgroups can harbor species with very different ploidy levels, e.g., the gamma-proteobacteria contain the polyploid species *Pseudomonas putida* and *Buchnera* spec. (20 genome copies and 120 genome copies, respectively) as well as *Escherichia coli*, which is monoploid during slow growth and mero-oligoploid during very fast growth in the laboratory (Pecoraro et al., 2011). In the group of alpha-proteobacteria, only one species has been analyzed until now, i.e., *Caulobacter crescentus*, which is monoploid under all conditions (Pecoraro et al., 2011).

Recently, three studies indicated that another alpha-proteobacterium, *Zymomonas mobilis*, might not be monoploid, but oligoploid or polyploid. Two studies with the strain Zm4 used transposon mutagenesis, and, surprisingly, found insertions in genes that were thought to be essential (Skerker et al., 2013; Brenac et al., 2019). Further analysis revealed that cells contained both native as well as transposon-interrupted copies, and, thus, were heterozygous and contained more than one genome copy. The formation of heterozygous cells upon attempts to inactivate essential genes have previously been described also for other groups of prokaryotes, e.g., cyanobacteria (Hubschmann et al., 1997; Spence et al., 2004; Takahama et al., 2004; Nodop et al., 2008), *Azotobacter vinelandii* (Suh et al., 2000), methanogenic archaea (Stock et al., 2010), and haloarchaea (Lange et al., 2011). Therefore, the heterozygocity of *Z. mobilis* is not a single exception. However, in all these cases a simultaneous high selection pressure for the presence of the marker used for gene inactivation as well as for the retention of the essential genes was applied, and, thus, the heterozygocity and oligo-/polyploidy might well have been experimental artifacts.

However, two observations in the absence of selection also indicate that *Z. mobilis* is oligo-/polyploid. In one of the above-mentioned studies the genome copy number was directly quantified using Real-Time-PCR, and it was found to be higher than 50 (Brenac et al., 2019). In addition, a very recent study with strain Zm6 used fluorescent microscopy to examine cell division and DNA content (Fuchino et al., 2021). It was revealed that the DNA content was heterogeneous in the population, implying that cells with a higher DNA content must contain more than one genome copy.

However, other results seem to contradict the idea that *Z. mobilis* is polyploid. For example, dozens of “clean mutants” have been generated, which at first sight seems to be impossible for a polyploid species (Kalnenieks et al., 2019b; Kosaka et al., 2020; Yang et al., 2020). Very recently, the development of CRISPR Interference System for the knock-down of gene expression in *Z. mobilis* has been described, and the species was designated as “possibly polyploid” (Banta et al., 2020).

We felt that these observations, discrepancies and uncertainties necessitated a systematic analysis of the ploidy level of *Z. mobilis*, because it is a species with very interesting physiology and high potential for biotechnological applications. The species can convert simple sugars to ethanol nearly at the maximal theoretical yield (Kalnenieks, 2006), with low incorporation of carbon source to its biomass (Swings and De Ley, 1977). Due to recent increasing environmental concerns, *Z. mobilis* has been intensively studied as a biocatalyst in a sustainable biorefinery system for fuel productions. Recently, attempts have been reported to modify its metabolism, with an emphasis to enhance its ability to convert abundant biomass into industrially valuable fuels (Wang et al., 2018). Several recent studies revealed that *Z. mobilis* cannot only be used for the production of ethanol, but also for the production of ethylene and various biopolymers (Li et al., 2020; He et al., 2021; Iftikhar et al., 2021). It could be shown that the production yields can be increased when cells are tolerant to phenolic substances (Yan et al., 2021; Yi et al., 2021). Several recent reviews summarize the metabolism of *Z. mobilis* and its application for various biotechnological applications (Kalnenieks et al., 2019a; Liu et al., 2019; Xia et al., 2019; Zhang et al., 2019; Kopp and Sunna, 2020; Todhanakasem et al., 2020). The characterization of the ploidy level and its regulation would not only deepen the insight into the cell biology of a second alpha-proteobacterium, but would also facilitate its biotechnological application.

Therefore, we characterized the genome copy number of three strains of *Z. mobilis* throughout the growth curves under the typical anaerobic conditions, which are accompanied by ethanol production. In addition, for one strain the genome copy number was also quantified during aerobic growth. The results finally clarified that *Z. mobilis* is polyploid, and they are compared to previous results with *Z. mobilis* and with other polyploid prokaryotes.

## Materials and Methods

### Bacterial Strains, Medium, and Growth Conditions

*Z. mobilis* strains Zm6 (ATCC29191) and Zm4 (ATCC31821) were obtained from the laboratory of Microbial and Cell Physiology (Professor Per Bruheim) at the Norwegian University of Science and Technology, Trondheim, Norway. The lab stock Zm6 is designated as Zm6N (Norway) in this study. These strains originate from the Laboratory of Microbial Bioenergetics at the University of Latvia. A strain with the designation Zm6 was also obtained from DSMZ (German Collection of Microorganisms and Cell Cultures), it had the strain collection name DSM 3,580. This strain is designated as Zm6G (Germany) in this study.

*Z. mobilis* was grown in the complex medium containing glucose (20 g/L), yeast extract (5 g/L), NH_4_SO_4_ (1 g/L), KH_2_PO_4_ (1 g/L), and MgSO_4_ (0.5 g/L). The complex medium was flashed with N_2_ gas prior to cultivation. 12 ml of anaerobic *Z. mobilis* culture was grown at 30°C in a capped 15 ml falcon tube with shaking at 200 rpm. For aerobic growth, 20 ml of culture in a 100 ml Erlenmeyer flask was shaken at 200 rpm, at 30°C.

To obtain a growth curves and collect samples, aliquots of exponentially growing pre-cultures were pelleted, resuspended and used to inoculate test cultures with a starting OD_600_ of 0.5. In each case, three biological replicates were performed. The OD_600_ was measured at the time points indicated in the respective growth curves using a Specord S600 spectrophotometer (Analytik Jena, Jena, Germany). Average values and their standard deviations were calculated. At the time points indicated in the respective growth curves, the aliquots were also used for the quantification of the genome copies per cell (see below).

### Strain Selection and Identification of the Replication Origin and Terminus

We aimed at a thorough characterization of the genome copy number of *Z. mobilis*, using several strains and quantifying the origin and terminus copy number. The strains Zm6 and Zm4 were chosen for the analysis, and the genome sequences of both strains had been reported previously (Seo et al., 2005; Desiniotis et al., 2012). The genome sizes are 1,961,306 bp for Zm6 and 2,056,363 bp for Zm4, respectively. The DoriC database (Luo and Gao, 2019) was used to identify the location of the replication origins, which were predicted to be at 1,353,901–1,354,938 nt for Zm6 and at 2,055,615–146 nt for Zm4 (numbering of the original genome sequence projects). For none of the two strains an experimental validation of these predicted *oriC* sites exist. However, several skew analyses (GC, AT, and MK disparities) as well as the presence of a cluster of DnaA binding sites are very strong indicators for the presence of the origin at the predicted site (Supplementary Figure 1). The replication terminus was predicted to be located opposite to the replication origin on the circular chromosome. Primers were designed to amplify regions of about 1 kbp adjacent to the predicted origin and terminus (Supplementary Table 1). The PCR fragments were used to generate standard curves for the copy number determination of the two sites in *Z. mobilis* cultures (see below).

### Optimization of Various Steps of the Real Time PCR Method for Its Application to *Z. mobilis*

**FIGURE 1:**
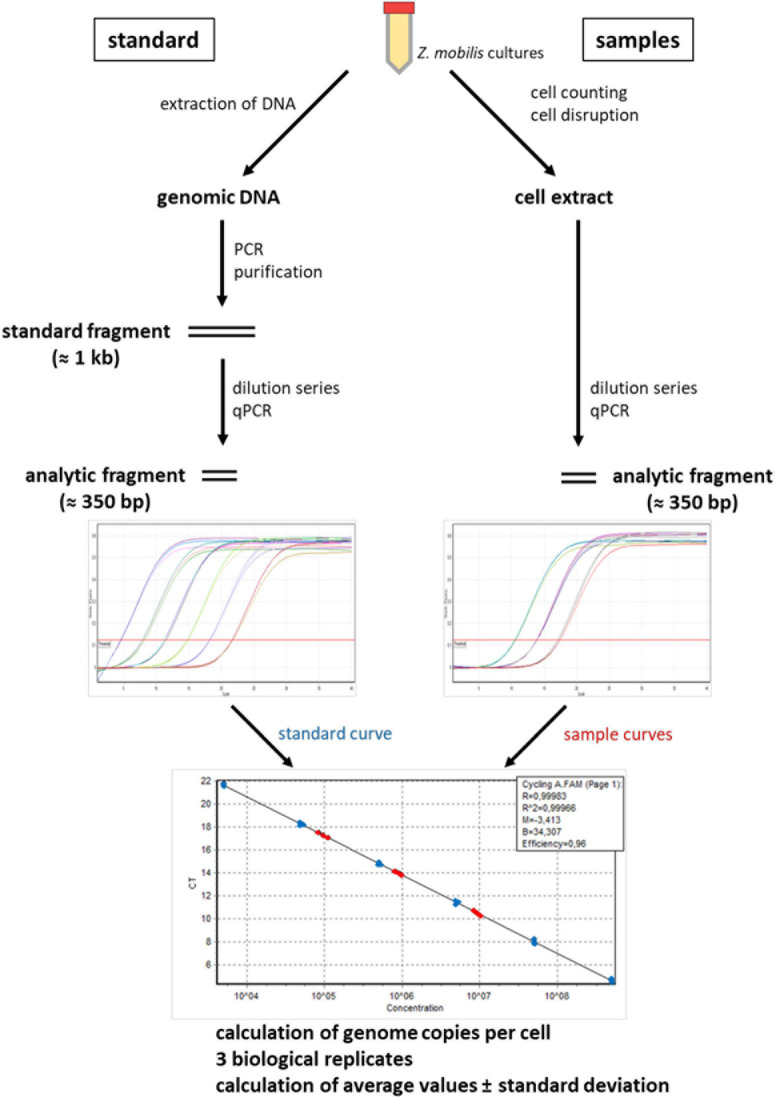
An overview of qPCR method to quantify genomic copy numbers in *Z. mobilis*.

Quantification of genome copy numbers via the Real-Time PCR methods is comprised of various steps, which are described in the next section. Figure 1 gives an overview of the whole experiment. Several steps have to be re-optimized for every new species, and the following steps were optimized for the application to *Z. mobilis*:

First, we optimized cell extraction method suitable for qPCR analysis. The requirements are that (nearly) 100% of all cells are disrupted, and that the genomic DNA stays mainly intact to enable quantitative analysis. The following five methods were tested:

(1) Shaking of cell suspensions with silica beads using a Speed mill. This methods turned out to be superior, and the details are included in the description of the optimized method below.
(2) The cells were treated with lysozyme (20 mg/ml in TE buffer) for 1 hour at room temperature, then 0.1% (w/v) SDS was added, and the suspension was shaken to disrupt cells.
(3) Cell suspensions were incubated with 3% (w/v) glycine at room temperature for 30 min. Then 1% (w/v) SDS was added to disrupt cells.
(4) Cell suspensions were incubated with 20 mg/ml (w/v) lysozyme for 1 h at 37°C. Then they were heated for 5 min to 95°C.
(5) Thawed cells were resuspended in 180 μl of TE buffer containing 20 mg/ml lysozyme, incubated at 37°C for 30 min. Then, 4 μl of protenaseK and 2 μl of 1% (w/v) SDS were added to the sample, followed by an incubation at 50°C for 30 min. Genomic DNA was isolated using the GeneJET DNA purification kit according to the instructions of the manufacturer (Thermo Fisher Scientific Inc., Waltham, United States).

Next, the analysis PCR fragments of about 350 nt were chosen (Figure 1). For the origin as well as for the terminus region two forward and two reverse primers were obtained and all possible combinations were tested using genomic DNA as template. The primer pairs that gave the best results are included in Supplementary Table 1.

Furthermore, we verified that the cytoplasmic extract of *Z. mobilis* did not contain any substances that reduced the exponential increase in product formation during the qPCRs. If PCR reactions are strictly exponential, a dilution of the template (standard fragment or cytoplasmic extract) by a factor of 10 must lead to a shift of the threshold value (ΔC_t_) by 3.32 cycles (log_2_ of 10). Tenfold serial dilutions of standard fragments and cytoplasmic extracts were generated and analyzed by qPCR. The ΔC_t_-values were close to 3.32, showing that all reactions were strictly exponential (examples are shown in the Supplementary Materials). Therefore, it was verified that the cytoplasmic extracts of *Z. mobilis* do not contain any substances that compromise the qPCR, and that they can be used for the quantification of genome copy numbers without any further treatment.

### Quantification of Genomic Copy Numbers Using the Optimized Real-Time-PCR Method

Quantification of genome copy numbers via the Real-Time PCR methods is comprised of various steps, which are described in the following sections. Figure 1 gives an overview of the whole experiment.

#### Generation of Standard Fragments

Genomic DNA of *Z. mobilis* was isolated using the GeneJET DNA purification kit according to the instructions of the manufacturer (Thermo Fisher Scientific Inc., Waltham, United States). It was used as template for the amplification of a standard fragments of about 1 kbp, which represented the regions near the replication origin and the replication terminus, respectively. The sequences of the primers are summarized in Supplementary Table 1. The regions were chosen so that identical primer pairs could be used for all three strains. Analytical agarose gel electrophoresis was used to verify that exclusively one fragment of the expected size was amplified. The fragment was purified using a PCR purification kit (New England Biolabs, Ipswich, MA, United States). Its mass concentration was quantified spectroscopically (1 OD_260_ = 50 ng/μl), its molar concentration was calculate using an average molecular mass of 660 g/Mol for one base pair, and the number of molecules per volume was calculated using the Avogadro number.

#### Isolation of Samples for the Analysis

*Z. mobilis* cultures for the quantification of genome copy numbers were grown as described above. At the time points indicated in the figures, the cell densities were quantified by cell counting using a Neubauer counting chamber (0.02 mm depth) and a light microscope (Axiostar Plus; Carl Zeiss, Oberkochen, Germany). 2–8 × 10^8^ cells were collected by centrifugation (2 min, 13,000 *g*). The supernatants were removed and the cell pellets were frozen at −80°C until they were analyzed.

#### Generation of Cell Extracts

Several methods for cell disruption were tested. The following method turned out to be superior for *Z. mobilis* and was routinely used:

The cell pellets were thawed and the cells were resuspended in 1 ml of TE buffer (10 mM Tris/HCl pH 8, 2.5 mM EDTA, 20 mM NaCl). The suspensions were transferred to 2 ml tubes that contained 0.5 g silica beads (0.1 mm; Roth, Karlsruhe, Germany). Cells were mechanically disrupted by 10 cycles of 30 s-beating by a Speedmill P12 (Analytik Jena, Jena, Germany). Cell disruption was performed in a cold room at 4°C to inhibit heating of the samples. Cell debris was removed by centrifugation, and the supernatant (cytoplasmic extract) was directly used for qPCR without any further treatment. The method was fast, reproducible, and the cell lysis efficiency was on average 84.2 ± 7.7% (*n* = 7). This efficiency was used for the calculation of genome copy numbers. It should be noted that occasionally “cell ghosts” were observed, i.e., cells that appeared much brighter in phase contrast microscopy. These were counted as not disrupted, because it was assumed that genomic DNA was probably retained even if most of the cytoplasmic content had leaked out. If these ghosts would be free of genomic DNA, the cell lysis efficiency would have been slightly underestimated.

#### Determination of Genome Copy Numbers Per Cell

**FIGURE 2:**
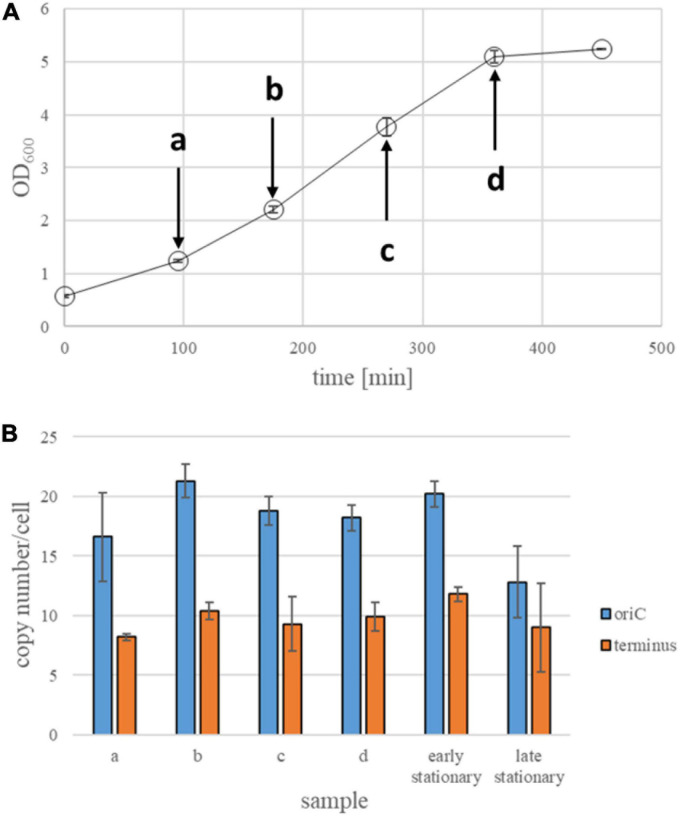
Quantification of origin and terminus copy numbers in strain Zm6N under anaerobic conditions. **(A)** Growth curves of Zm6N strain under anaerobic conditions. Average OD_600_ values of three biological replicates and their standard deviations are shown. Arrows indicate time points of collecting samples for quantification of genomic copy numbers. **(B)** Origin and termini copy numbers in anaerobically growing Zm6N. Samples at an early stationary phase were collected about 1 h after the culture reached a plateau of its growth curve. Samples at late stationary phase were collected abo8 h after the culture hit the plateau. All time points at growth phase are three biological replicates, while two biological replicates at an early stationary phase and 5 biological replicates were made for a late stationary phase. Standard deviations (STD) are presented as error bars.

**FIGURE 3:**
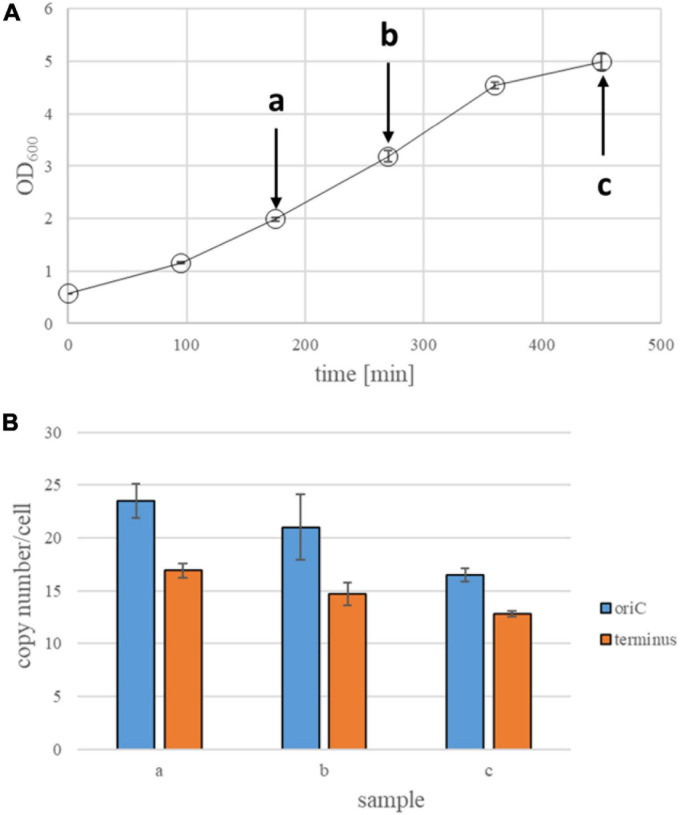
Quantification of origin and terminus copy numbers in strain Zm6G under anaerobic conditions. **(A)** Growth curves of Zm6G strain under anaerobic conditions. Average OD_600_ values of three biological replicates and their standard deviations are shown. Arrows indicate time points of collecting samples for quantification of genomic copy numbers. **(B)** Origin and termini copy numbers in anaerobically growing Zm6G. Samples at an early stationary phase were collected about 1 h after the culture reached a plateau of its growth curve. All time points at growth phase are three biological replicates, while two biological replicates at an early stationary phase and 5 biological replicates were made for a late stationary phase. Standard deviations (STD) are presented as error bars.

**FIGURE 4:**
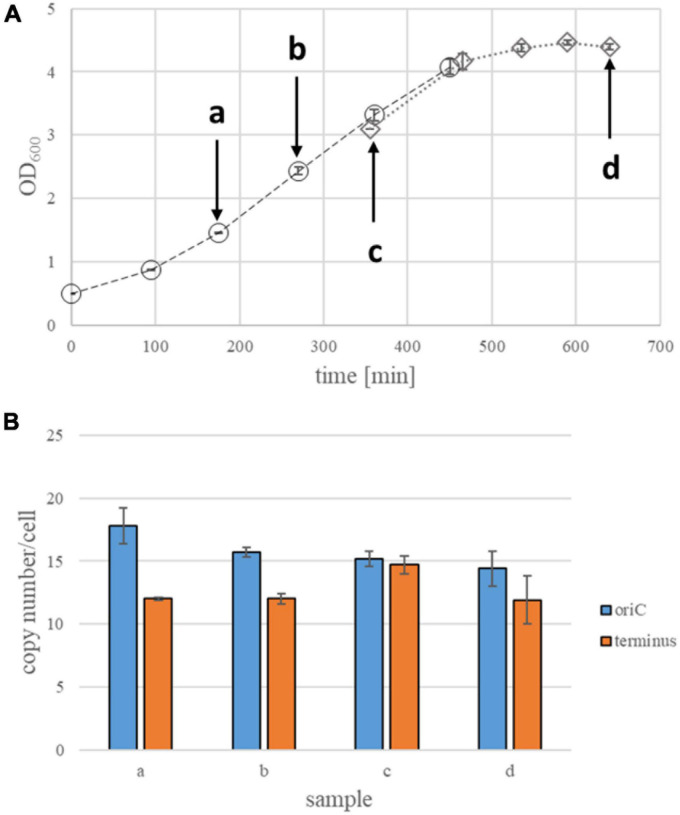
Quantification of origin and terminus copy numbers in strain Zm4 under anaerobic conditions. **(A)** Growth curves of Zm4 strain under anaerobic conditions. Average OD_600_ values of three biological replicates and their standard deviations are shown. Two sets of experiments (three biological replicates each) were performed, because in the first set of experiments the results in stationary phase did not meet the quality criteria (see different symbols and different dashed lines). Arrows indicate time points of collecting samples for quantification of genomic copy numbers. **(B)** Origin and termini copy numbers in anaerobically growing Zm4. Samples at an early stationary phase were collected about 1 h after the culture reached a plateau of its growth curve. All time points at growth phase are three biological replicates, while two biological replicates at an early stationary phase and 5 biological replicates were made for a late stationary phase. Standard deviations (STD) are presented as error bars.

**FIGURE 5:**
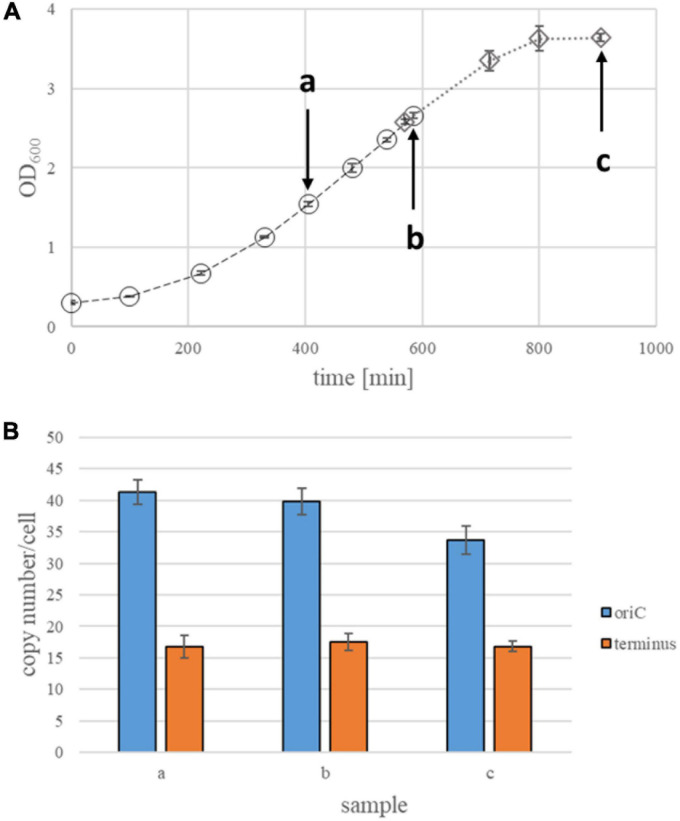
Quantification of origin and terminus copy numbers in strain Zm6N under aerobic conditions. **(A)** Growth curves of Zm6N strain under aerobic conditions. Average OD_600_ values of three biological replicates and their standard deviations are shown. Two sets of experiments (three biological replicates each) were performed, because in the first set of experiments the results in stationary phase did not meet the quality criteria (see different symbols and different dashed lines). Arrows indicate time points of collecting samples for quantification of genomic copy numbers. **(B)** Origin and termini copy numbers in aerobically growing Zm6N. Samples at an early stationary phase were collected about 1 h after the culture reached a plateau of its growth curve. All time points at growth phase are three biological replicates, while two biological replicates at an early stationary phase and 5 biological replicates were made for a late stationary phase. Standard deviations (STD) are presented as error bars.

A dilution series of the standard fragment from 10^–3^ to 10^–8^ was generated. The cytoplasmic extracts were diluted 10- and 100-fold. Three technical replicates were analyzed for every dilution, and three technical replicates of a “no template control” were included. All samples were analyzed simultaneously by Real Time PCR using a RotorGene 3000. The C_t_ values of all samples were determined, and it was verified that the ΔC_t_ values between the 10-fold dilution steps were around 3.32. The average C_t_ values of the standard dilutions were used to generate a standard curve, which was used to determine the origin and terminus copy numbers present in the dilutions of the cytoplasmic extracts. Together with the disrupted cell numbers used for cell extract generation, these values were used to calculate the copy numbers per cell. Three biological replicates were performed for each strain/growth condition. At least two dilutions of the cytoplasmic abstracts could be analyzed, therefore six technical replicates were used for every biological replicate. Average values and their standard deviations were calculated, and are summarized in Figures 2B– 5B.

### Analysis of Cell Morphology and Quantification of Cell Volumes

**FIGURE 6:**
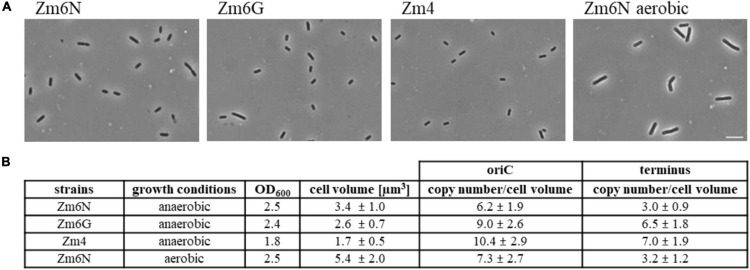
Determination of genome copy numbers per μm^3^ cell volume. **(A)** Phase contrast images of anaerobically growing Zm6N, Zm6G, and Zm4, and aerobically growing Zm6N cells. Scale bar; 7.85 μm. **(B)** A table of cell volume and genomic copy numbers per cell volume in Zm6N, Zm6G, and Zm4 during anerobic growth, and Zm6N during aerobic growth. Cell length and width were measured and used for calculating approximate cell volume. Sample numbers for cell length and width measurements; *N* = 110 and 53 for anaerobically growing Zm6N, respectively, *N* = 120 and 52 for Zm6G, *N* = 110 and 57 for Zm4, and *N* = 120 and 56 for aerobically growing Zm6N.

The *Z. mobilis* strains were cultivated as described above, and samples for the cell volume determination were taken at the same time points as those for genome copy number quantification. An Axioskop 40 light microscope (Carl Zeiss, Oberkochen, Germany) equipped with a AxioCam MRm camera was used for capturing phase contrast images of the cells. Lengths and widths of *Z. mobilis* cells were manually measured using software AxioVision Rel. 6 (Carl Zeiss, Oberkochen, Germany). Note that we occasionally observed chained cells, i.e., daughter cells that had completed septation but were not yet separated from each other. Each chained cell was measured as individual single cell, as we were interested in copy numbers per single cell volume. Average values of cell length and cell width were used to calculate average cell volumes and their standard deviations. The cell shape was approximated as a cylinder for cell volume calculation. The numbers of measured cells are included in the legend of Figure 6.

## Results

### Identification of the Locations of Replication Origin and Terminus in the Genome of *Z. mobilis*

In slowly growing species, all sites of the genome have an identical copy number. However, during fast growth, the number of replication origins can be considerably higher than the number of replication termini. For example, cultures of the gram-negative gamma-proteobacterium *Escherichia coli* that grow with generation times of 25 min contain, on average, 6.8 origins and 1.7 termini (Pecoraro et al., 2011). This phenomenon has been named mero-oligoploidy, and it is a pre-requisite for fast growth. We aimed at quantifying the genome copy number of the alpha-proteobacterium *Z. mobilis*. For a thorough analysis, it was decided (1) to analyze several strains, and (2) to determine the copy numbers of both replication origin and replication terminus, and, thereby, include the clarification of whether they are identical or whether the origin copy number is higher than the terminus copy number. The selection of strains and the prediction of the localizations of origin and terminus are described in the Methods section.

### The Real-Time-PCR Method for the Quantification of Genome Copy Numbers and Its Optimization for the Application With *Z. mobilis*

The Real-Time-PCR (qPCR) method has been developed in our laboratory for the quantification of genome copy numbers in halophilic archaea (Breuert et al., 2006). Since then, it has been successfully applied for the copy number quantification in various groups of prokaryotes, including several species of proteobacteria (Griese et al., 2011; Hildenbrand et al., 2011; Pecoraro et al., 2011; Böttinger et al., 2018). It has been validated with complementary approaches (quantitative Southern blotting, spectroscopy analyses), and the results were in full agreement with previous knowledge for well-studied species like *E. coli* (Pecoraro et al., 2011). Figure 1 gives an overview of the qPCR method. For every new species, several steps have to be re-established and, if necessary, optimized. The optimization of several steps for the application of the method to *Z. mobilis* are described in detail in the Methods section.

### Determination of Ploidy Levels in the Strain Zm6N Under Anaerobic Conditions

First, the genome copy numbers of the strain Zm6N during anaerobic growth in complex medium were determined. Figure 2A shows a growth curve, the doubling time in exponential phase was 99.7 min. Aliquots for qPCR analysis were removed at the four time points indicated by arrows as well as during early and late stationary phase. The results are summarized in Figure 2B. The origin copy number was rather constant from early exponential to early stationary phase and had an average value of 19.0, clearly showing that *Z. mobilis* Zm6N is polyploid under anaerobic conditions throughout growth. However, during late stationary phase the copy number slightly decreased to a value of 12.8. This decline in origin copy number in stationary phase is highly significant (*t*-test, *p* = 0.0012).

The copy number of the terminus region was considerably lower, with an average value of 9.9 from early exponential to early stationary phase. This meets the expectation, because in a non-synchronized culture growing with a doubling time that is shorter or not much longer than the replication plus segregation time, there is always a considerable fraction of the population that has started replication, and, thus, contains twice as much origins than termini. The number of termini is not downregulated in late stationary phase, so that the numbers of origins and termini became more similar. In fact, the standard deviation revealed that they are not significantly different, indicating that replication has ceased in late stationary phase.

Taken together, the results revealed that the strain Zm6N contains nearly 20 origins and 10 termini, and that the numbers are not differentially regulated throughout growth, in contrast to other polyploid bacteria and archaea (see section “Discussion”).

### Determination of Ploidy in Zm6G Cells Under Anaerobic Conditions

Prokaryotic species can accumulate mutations upon prolonged cultivation in the laboratory. For example, two strains of *H. salinarum*, which originated from one isolate, have experienced major rearrangements in their minor chromosomes, so that number and sizes of the minor chromosomes differ and each strain contains genetic information that is missing from the other (Pfeiffer et al., 2008). Another example is the cyanobacterium *Synechocystis* PCC 6803, for which six substrains with slightly different phenotypes have been described (Zerulla et al., 2016). Also for the *Z. mobilis* strain Zm6N several mutations have been observed (Fuchino and Bruheim, 2020). Because these mutations might possibly have an influence the genome copy number, another Zm6 strain was retrieved from the German Collection of Microorganisms and Cell Cultures.^1^ This strain, designated Zm6G in this study, was also grown under anaerobic conditions in complex medium, and analyzed as described above. Figure 3A shows the growth curve, the strain had a generation time of 108.3 min during exponential growth, very similar to the strain Zm6N. The results of the copy number determination are shown in Figure 3B. Strain Zm6G had, on average, 22.3 origin copies during exponential growth very similar to strain Zm6N. The origin copy number was already somewhat lower at early stationary phase (16.5), indicating that the copy number decrease might start earlier in Zm6G than in Zm6N. The difference is statistically significant (*t*-test, *p* = 0.0086). However, this decline rests on a single time point, and, thus, this might not be a real difference, in spite of the quantification of three biological replicates (with six technical replicates each) and low standard deviations.

However, the terminus copy number is clearly higher in strain Zm6G (on average 14.8 from early exponential to early stationary phase) than in strain Zm6N (on average 9.9, see above). The origin/terminus ratios were 1.5 for strain Zm6G vs. 1.9 for stain Zm6N, indicating that the two strains might have differences in the replication or segregation times (see section “Discussion”). However, both Zm6 strains were found to be polyploid and have very similar numbers of replication origins during anaerobic growth.

### Determination of Ploidy in the Strain Zm4 Cells Under Anaerobic Conditions

A genome comparison of eight substrains of *Z. mobilis* revealed a conserved core backbone of the genome, but also various mutations that had occurred during species evolution (Chen et al., 2018). A phylogenetic tree was constructed based on the whole genome sequences (compare Figure 3 in Chen et al., 2018). In this tree, strains Zm6 and Zm4 had the largest possible phylogenetic distance, therefore, Zm4 was chosen as the second strain for this study.

In comparison to the other strains, Zm4 has two large genomic inversions (Chen et al., 2018). Zm4 has a higher number of genes than Zm6 (1,819 vs. 1,787) and a higher number of plasmids. Zm6 contains three plasmids, while Zm4 contains four or five plasmids (Chen et al., 2018; Yang et al., 2018). There are also phenotypic differences, e.g., the Zm4 strain exhibits motility (Seo et al., 2005) and a floc formation (Jones-Burrage et al., 2019), in contrast to Zm6, while Zm6 is superior to other strains in levan production (Chen et al., 2018). In addition, we observed that cell size of Zm4 was significantly smaller than that of Zm6 cell (see volume determination below).

Strain Zm4 was also grown anaerobically in complex medium, and the growth curve is shown in Figure 4A. The doubling time during exponential phase was 117.5 min, slightly larger than strain Zm6. The genome copy number was quantified at three time points during exponential growth and at early stationary phase, and the results are summarized in Figure 4B. The average origin copy number was 15.8, about 20–30% lower than in the two Zm6 strains. The average terminus copy number was 12.7, and, thus, the origin/terminus ratio was 1.2, the lowest value of the three strains.

### Verification of Strain Differences

The results presented above were based on three biological replicates, each with six technical replicates, and were, therefore, very well supported and had low standard deviations. Nevertheless, two further experiments were performed to verify that the observed strain differences were real and not due to stochastic variances. First, further three biological replicates of each strain were grown to mid-exponential phase (OD_600_ around 2), and the origin and terminus copy numbers were quantified. The results of the growth curves (Figures 2–4) as well as the new results are summarized in Table 1. The new results were very similar to the first results and underscored that strain differences exist, e.g., that Zm4 has a lower origin number than the other two strains, and that Zm6N has the highest origin/terminus ratio.

**TABLE 1:** Summary of quantifications of origin and terminus copy numbers.

**Strain**	**Zm6N**	**Zm6G**	**Zm4**
**Origin copy number**			
Growth curves (Figures 2–4)	19.0 ± 1.7	22.3 ± 2.4	15.8 ± 1.0
Exponential growth	18.4 ± 2.1	22.7 ± 2.3	15.6 ± 0.7
**Terminus copy number**			
Growth curves (Figures 2–4)	9.9 ± 1.0	14.8 ± 0.7	12.7 ± 0.8
Exponential growth	7.9 ± 0.8	17.0 ± 0.8	13.8 ± 1.3
**Ratio origin/terminus**			
Growth curves	1.9	1.5	1.2
Exponential growth	2.3	1.3	1.1

*The table summarizes the average values that were obtained from early exponential to early stationary phase (“growth curves”) and results from additional control cultures that were grown to mid-exponential growth phase (“exponential phase”).*

In a second control experiment, the termini copy numbers in exponentially growing cultures were quantified using two different pair of oligonucleotides (Supplementary Tables 1, 3). It was verified that the terminus copy number in Zm6G was higher than in Zm4, irrespective of the primer pair used for the analysis. Taken together, two control experiments confirmed the results of the growth curves, particularly, that differences between the three strains exist.

### Determination of Ploidy in the Strain Zm6N Cells Under Aerobic Conditions

Next, we aimed to unravel whether environmental conditions have an influence on the ploidy level, specifically, whether aerobic conditions change the ploidy level. Under aerobic conditions, *Z. mobilis* produces acetaldehyde, which is toxic to the cells and perturbs growth (Wecker and Zall, 1987; Kalnenieks et al., 2019b). As acetaldehyde is a valuable compound for industrial applications, *Z. mobilis* strain Zm6 had previously been engineered for an efficient production of acetaldehyde (Kalnenieks et al., 2019b).

Strain Zm6N was grown under aerobic conditions and a growth curve was recorded (Figure 5A). As expected, strain Zm6N grew considerably slower under aerobic conditions. The doubling time during exponential growth was 187.3 min (instead of 99.7 under anaerobic conditions), and it took 800 min until stationary phase was reached (instead of 350 min). Origin and terminus copy numbers were quantified at two time points during exponential growth and at early stationary phase (Figure 5B). In spite of the slow growth, the origin copy numbers were much higher than during fast, anaerobic growth (about 40 vs. about 20). Similarly, the termini numbers were also higher (around 17 vs. around 9). However, we observed that the aerobically growing cells exhibited an elongated cell shape. This is in accordance with previous knowledge, because it has been reported that an elongated cell shape of *Z. mobilis* is induced by stress conditions like high temperature or elevated salt concentration (Vriesekoop et al., 2002; Hayashi et al., 2012; Fuchino and Bruheim, 2020).

### Genomic Copy Numbers Per Cell Volume

The observations that both genome copy numbers as well as cell length of strain Zm6N were elevated during aerobic growth led to the question whether the copy number per cell volume might be regulated and constant. To this end, microscopic pictures of all three strains during anaerobic growth and of Zm6N during aerobic growth were taken (Figure 6A). Cell lengths and cell widths were measured, and average values were used to calculate average cell volumes (Figure 6B). Then, the origin and terminus copy numbers reported above were normalized to the cell volume (Figure 6B). The about twofold difference between copy numbers of Zm6N during aerobic and anaerobic growth was largely diminished after normalization, i.e., the difference was less than 20% for the origin copy number, and the terminus copy numbers were nearly identical. These results indicate that the copy number regulation in Zm6N is sensitive to the cell size, but is not influenced by the growth rate.

The average cell volumes of strains Zm6G and Zm4 differed from that of Zm6N, i.e., Zm6G was 14% smaller, while Zm4 had only half the volume of Zm6N. Interestingly, ploidy level differences persisted even after normalization to the cell volume. Strain Zm4 exhibited the highest genome density, and Zm6N the lowest genome density (Figure 6B). Taken together, we observed strain differences concerning the average cell volume as well as the genome density, in addition to the strain differences previously reported in the literature and the differences reported above.

## Discussion

In this study we have shown that three strains of *Z. mobilis* are polyploid (Zm6N, Zm6G, and Zm4). It should be noted that only a single method—quantitative Real Time PCR—was applied to reach this conclusion. However, the method had been extensively benchmarked against other methods as well as against other species in previous studies. As we established the method, it was benchmarked against quantitative Southern blotting (Breuert et al., 2006). In another study, it was benchmarked against spectroscopic quantification of genome copy numbers (Hildenbrand et al., 2011). In our first study with bacteria, the origin and terminus copy numbers of fast-growing as well as slow-growing *E. coli* cells were quantified, thereby the method was benchmarked against the wealth of information about *E. coli* copy number obtained by other groups with other methods (Pecoraro et al., 2011). In addition, origin and terminus copy numbers of the gram-positive model bacterium *Bacillus subtilis* were determined (Böttinger et al., 2018). In summary, the method has been benchmarked extremely thoroughly in previous studies, and, therefore, we did not benchmark it again in this study with *Z. mobilis*.

The observed polyploidy of *Z. mobilis* is in accordance with previous observations that heterozygous cells exist (at least upon double laboratory selection), which cannot be monoploid (Skerker et al., 2013; Brenac et al., 2019). In one study the copy number of the strain Zm4 had been quantified, and it was reported that it is between 60 and 90 (Brenac et al., 2019). These values are much higher than the around 16 origins and 13 termini per cell that we detected. A reason for this large difference is not obvious, because Brenac et al. (2019) used the same Real Time PCR method for copy number quantification that was applied in this study, and the culture conditions were similar. While the quantitative difference of the results cannot be explained, both studies agree that *Z. mobilis* Zm4 is a polyploid alpha-proteobacterium.

*Z. mobilis* is only the second alpha-proteobacterium for which the genome copy number has been quantified. The other species is *Caulobacter crescentus*, which is a monoploid bacterium under all conditions (Pecoraro et al., 2011). Severe changes in DNA content have been reported for nitrogen-fixing symbiotic alpha-proteobacteria (*Rhizobium, Bradyrhizobium*). However, in these cases the high DNA content is confined to the differentiated bacteriods within the root nodules of plants (Kondorosi et al., 2013; Maróti and Kondorosi, 2014). In fact, the differentiation to nitrogen-fixing bacteriods is enforced by a large number of peptides that are produced by the plants. To our knowledge, the ploidy level of free-living cells of *Rhizobium*/*Bradyrhizobium* has never been quantified, but the more than 20-fold difference in DNA content indicates that they might well be monoploid.

The three strains of *Z. mobilis* were found to have a constant number of chromosomes from early exponential to early stationary growth phase (Figures 2–5). This is different in other polyploid prokaryotes. For example, in haloarchaea the copy number increases from early to mid-exponential phase, and it decreases again at the end of the exponential growth phase (Breuert et al., 2006). The same is true for the methanogenic archaeon *Methanococcus maripaludis* (Hildenbrand et al., 2011). A severe down-regulation of the copy number during growth has also been reported for the cyanobacterium *Synechocystis* PCC 6803 (Zerulla et al., 2016). However, regulation of the ploidy level throughout the growth curve has been determined only for very few species, therefore, it is unclear whether a constant chromosome number or differential regulation is more widespread.

In addition to the previously known strain differences between Zm6 and Zm4 (Chen et al., 2018), additional differences were detected in this study, and, furthermore, it was found that also two Zm6 strains from different sources exhibit slight differences. One difference was the number of origins, which was considerably lower in Zm4 than in the two Zm6 strains. Even more surprising was the difference in the origin/terminus ratio, which was only 1.1 in Zm4, but about 2.1 in Zm6N (Table 1). The number of origins and termini have been quantified for *E. coli* cultures with five different growth rates, with doubling times from 24 to 100 min (Bremer and Dennis, 1996). The faster *E. coli* grows, the higher is the origin/terminus ratio, e.g., it is 1.6 for cells with a doubling time of 100 min, 2.2 for cells with a doubling time of 40 min, and 3.4 for a doubling time of 24 min. This is logical, because the initiation of replication has to be strictly coupled to the doubling time, otherwise the chromosome number would not be constant. However, the doubling times of the three *Z. mobilis* were very similar, and, thus, this cannot explain the twofold difference in the origin/terminus ratio. One possible explanation could be that the replication speed and thus the duration of the S-phase are different. The numbers of origins and termini are identical in the G1 and G2 phase, and, therefore, the origin/terminus ratio is smaller when the S-phase takes only a small fraction of the cell cycle, and larger when it takes a large fraction of the cell cycle. It will be interesting to analyze whether the cell cycle parameters of the three strains indeed differ, or whether another explanation for the difference of the origin/terminus ratio has to be found.

It was observed that the three strains have different sizes during exponential growth under identical conditions, therefore, the cell volumes and the origin and terminus density per μm^3^ were calculated (Figure 6). The genome density was highest for the smallest strain, Zm4, and it was considerably lower in Zm6N (9.3 vs. 5.6 origins and 7.5 vs. 2.9 termini per μm^3^). A comparison is not easy, because genome densities have only very rarely been reported. For *E. coli* growing with a doubling time of 99 min in synthetic medium on glycerol, a density of 1 origin (and 1 terminus) per μm^3^ has been reported (Wang et al., 2011). For *B. subtilis* growing in complex medium, a density of 1.4 genomes per μm^3^ has been reported, while for the giant bacterium *Epulipiscium* with many thousand genome copies a genome density of 0.5 per μm^3^ has been determined (Mendell et al., 2008). It seems that for normal-sized bacteria the genome density is higher in polyploid than in monoploid species, while this does not hold true for very large polyploid bacteria.

The proof that *Z. mobilis* is polyploid opens a variety of novel research questions that cannot be analyzed with monoploid species. For example, very recently it was shown that *Z. mobilis* divides asymmetrically and that the DNA content of the daughter cells is not identical, but heterogeneous. Such a relaxation of the strict regulation of septum formation at mid-cell as well as the segregation of identical DNA amounts to both daughter cells has first been observed for the methanogenic archaeon *Methanocaldocossus jannaschii*, which forms daughter cells that are extremely different in cell size and DNA content (Malandrin et al., 1999). To a much lesser extent, an uneven DNA distribution has also been described for the polyploid cyanobacterium *Synechocystis* PCC 6803 (Schneider et al., 2007). It remains to be analyzed whether an uneven DNA distribution to daughter cells might be more widespread among polyploid prokaryotes.

A further question is whether or not *Z. mobilis* has the normal cell cycle phases, specifically, whether it has an S-phase. For the oligoploid cyanobacterium *Synechococcus elongatus* it has been shown that at any given time only one out of five genome copies is replicated, while all copies are transcribed (Ohbayashi et al., 2019). Also for the polyploid haloarchaeon *Halobacterium salinarum* it was shown that replication is evenly spread over the whole cell cycle, and an S-phase is absent (Zerulla et al., 2014). Replication of a different number of genome copies at any given time in the three *Z. mobilis* strains could be an alternative explanation to the differences discussed above. Many additional features of polyploidy in prokaryotes have been discussed (Soppa, 2014; Ludt and Soppa, 2019), which can now also be studied with *Z. mobilis*.

## Conclusion

A very thorough analysis of the ploidy level of *Z. mobilis* is presented. In particular, the origin copy numbers and termini copy numbers were quantified in three strains throughout the whole growth curve during anaerobic growth. In addition, the copy number were also quantified for one strain during the for *Z. mobilis* unfavorable aerobic growth. For all strains under both conditions it was shown that *Z. mobilis* is polyploid, and, thereby, a pertinent discussion in the field has been settled.

Notably, strain-specific differences were observed concerning cell size, numbers of origins and termini, and the origin/terminus ratio. These results open research fields that are only relevant for polyploid prokaryotic species, in contrast to monoploid species. In addition, the results will be relevant for the further optimization of *Z. mobilis* for biotechnological applications.

## Data Availability Statement

The original contributions presented in the study are included in the article/Supplementary Material, further inquiries can be directed to the corresponding author/s.

## Author Contributions

KF and JS designed the project and wrote the manuscript. KF performed the experiments. KF, DW, and JS analyzed the data, edited, and revised the manuscript. All authors contributed to the article and approved the submitted version.

## Conflict of Interest

The authors declare that the research was conducted in the absence of any commercial or financial relationships that could be construed as a potential conflict of interest.

## Publisher’s Note

All claims expressed in this article are solely those of the authors and do not necessarily represent those of their affiliated organizations, or those of the publisher, the editors and the reviewers. Any product that may be evaluated in this article, or claim that may be made by its manufacturer, is not guaranteed or endorsed by the publisher.

## Footnotes

1 www.dsmz.de, strain designation DSM3580.
