# Supplementary material for: Genome Copy Number Quantification Revealed That the Ethanologenic Alpha-Proteobacterium Zymomonas mobilis Is Polyploid
Source: Front Microbiol. 2021 Aug 2;12:705895. doi: 10.3389/fmicb.2021.705895 (PMC8365228; doi:10.3389/fmicb.2021.705895)
Supplement: Supplementary file 1 [file Data_Sheet_1.docx]

**SUPPLEMENTARY MATERIAL**


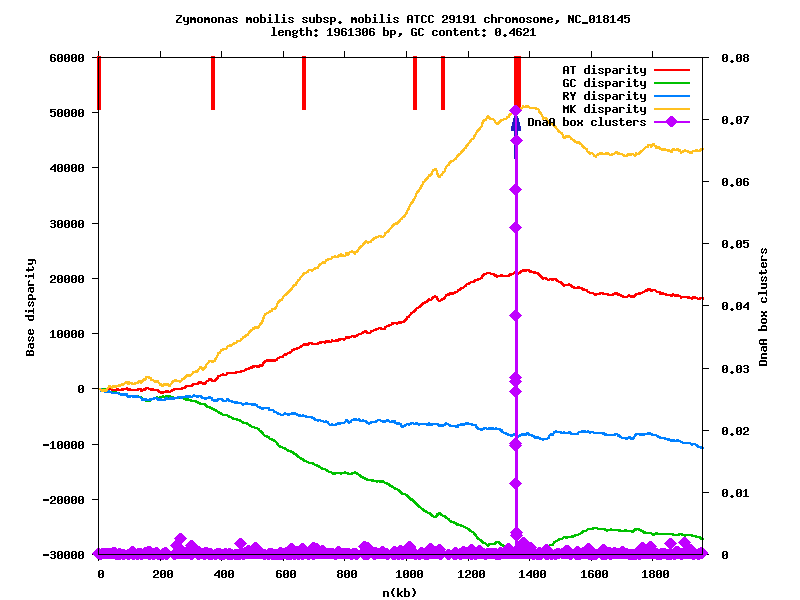


**Supplementary Figure S1**. GC/AT skew profiles and DnaA box prediction in the genome in ATCC29191 (Zm6). The profile was obtained from the DoriC database [Luo 2019]. The region with the lowest GC skew contains abundant DNA box clusters, suggesting the *oriC* site.

**Supplementary Table S1**. A list of primer used in the study.

| OriC/Terminus | Purpose | Primer name | sequence |
| --- | --- | --- | --- |
| OriC | Standard Fragments | oriF1 | TTCGTGATCGGCCATTGGTTTC |
|  |  | oriR2 | CAATGACCATTTCGGCCACCTG |
|  | qPCR analysis | oriqF3 | ATTACGCAAGCAAGACCTCC |
|  |  | oriqR3 | ACGGACATTATCGGGATTGTCG |
| Terminus | Standard Fragments | TerF1 | ATCGCCATGCCCTGACTGTG |
|  |  | TerR1 | AGGGCAGGATTTCGGCATGG |
|  | qPCR analysis | TerqF3 | CTGGCTCACAGCATCGAAAC |
|  |  | TerqRv4 | GAGGGCGGCTATTTGAGGAC |
|  | qPCR analysis | TerqF1 | CAAACCGATTGGCTTGAAGG |
|  |  | Terqrv3 | AAGGGACGACTGATAGTGGC |

**Supplementary Table. S2**. Ct and ΔCt profiles of Real-time quantitative PCR analysis. ΔCt between a sample and the 10 times diluted sample should be about 3.32, if the reaction was exponential, as explained in the main text. Reasonably, similar value to 3.32 was observed in reactions using Standard Fragments or *Z. mobilis* cell extracts as templates. NTC stands for non-template control.

**Supplementary Table S3**. Termini copy numbers in anaerobically growing *Z. mobilis* strains, using two different primer sets from the analysis in the Figure2-5. The presented copy numbers are an average of 6 technical replicates. R1 and R2 stands for biological replicate numbers. Standard deviation is presented as an error.
